# A multi-granularity narrative cloud selection framework for architectural meaning-making integrating the PIA model and three-way decision theory

**DOI:** 10.3389/fpsyg.2026.1869490

**Published:** 2026-10-06

**Authors:** Qi Li

**Affiliations:** School of Fine Arts, Shanxi University, Taiyuan, China

**Keywords:** architectural meaning-making, granular computing, human-centered sustainability, narrative cloud, neuroaesthetics, three-way decision

## Abstract

Architectural experience depends not only on perceptual fluency but also on whether users can successfully construct higher-order semantic meaning from complex built form. In the Pleasure-Interest Model of Aesthetic Liking (PIA model), the controlled-processing stage differentiates interest, confusion, and boredom. In conventional architectural cognition research, however, confusion is often treated as a negative indicator of communicative failure. This paper challenges that assumption and argues that confusion is especially important in architectural meaning-making because it identifies a population that still possesses interpretive motivation but has not yet achieved semantic resolution due to a mismatch between narrative granularity and cognitive accessibility. To address this problem, the paper proposes a conceptual and heuristic framework that integrates the PIA model, three-way decision theory, and granular computing. First, the three states in the controlled-processing stage of the PIA model are reinterpreted through a three-way decision structure, in which confusion is reconstructed as a strategic boundary region rather than as a purely negative endpoint. Second, a multi-granularity optimization mechanism and a utility-based selection function are introduced to identify narrative descriptions that balance novelty and comprehensibility for target perceivers. Third, a memorial-hall case and a computer-simulated illustrative statement-scoring exercise are used to illustrate the feasibility of the proposed procedure. Rather than claiming full empirical validation, the paper positions its contribution as a design-oriented framework for improving architectural communication, user-centered narrative intervention, and the cultural-social dimension of human-centered sustainability in the built environment.

## 1 Introduction

Recent scholarship in neuroaesthetics, architectural cognition, and human-building interaction has clarified that built environments are not merely visual objects but meaning-bearing settings that shape perception, affect, behavior, and interpretation through layered interactions among sensory, cognitive, and contextual processes (Pearce et al., 2016; Coburn et al., 2017; Chatterjee and Vartanian, 2014; Alavi et al., 2019). At the same time, recent studies in architecture and the built environment increasingly emphasize visual preference, restorative response, public interpretation, virtual perception, and user-centered sustainable design as interconnected issues rather than isolated topics (Liu and Guo, 2024; Son and Kim, 2024; Deng et al., 2026; Sedghikhanshir and Montelli, 2026; Wu et al., 2024; Ji and Li, 2024; Bunyarittikit et al., 2026). These developments suggest that architecture should be studied not only as form, but also as a medium through which meaning is organized, communicated, and experienced.

One useful framework for understanding this process is the Pleasure-Interest Model of Aesthetic Liking (PIA model) (Graf and Landwehr, 2015). On the one hand, the PIA model captures the richness of aesthetic preference by distinguishing low-level automatic processing from high-level controlled processing. On the other hand, it explains why the same object may produce different aesthetic outcomes depending on whether the perceiver is willing and able to invest cognitive effort in resolving novelty, ambiguity, or complexity. In this sense, the PIA model reconciles the apparent conflict between fluency-based preference (Reber et al., 2004) and findings showing that complexity, novelty, and challenge may also support positive aesthetic evaluation when they can eventually be interpreted (Papadimitriou, 2020; Van de Cruys et al., 2021; Song et al., 2021; Minissale, 2013; Güçlütürk et al., 2016).

The concept of fluency has long occupied a central place in aesthetic theory. Derived from experimental aesthetics and later developed in cognitive psychology, fluency has important implications for preference, familiarity, and judgments of meaning (Zajonc, 1980; Winkielman et al., 2006; Hassan and Barber, 2021; Whittlesea, 1993). In the controlled-processing stage of the PIA model, three states are especially important: interest, confusion, and boredom. From the perspective of architectural meaning-making, confusion is particularly significant because it captures a condition in which perceivers are motivated to understand a design but remain unable to complete interpretation. In architecture, this condition is not trivial. Buildings are often appreciated for reasons that exceed pure beauty, including cultural symbolism, semantic associations, experiential narratives, and the social meaning embedded in space (Jo and Lee, 2007; Achten, 2019).

This issue becomes more salient when architecture is approached as narrative. Narrative architecture may be understood as a design strategy through which buildings communicate stories, values, symbolic references, and semantic cues by means of form, spatial sequence, material expression, and atmosphere (Jo and Lee, 2007; Achten, 2019). In this process, architecture is not only perceived; it is also read, inferred, and interpreted. However, different perceivers bring different cultural knowledge, professional backgrounds, and perceptual expectations. As a result, the same architectural intention may produce very different interpretive outcomes. If the selected narrative discourse is too fine-grained, highly abstract, or dependent on specialized background knowledge, users may remain in confusion rather than progressing toward meaningful engagement; if it is too coarse and overly explicit, the architectural narrative may become readable but lose semantic tension and experiential value.

To address these issues, this paper proposes a multi-granularity narrative cloud selection framework integrating the PIA model, three-way decision theory, and granular computing. The central claim is that confusion should not be treated as a purely negative result. Instead, it can be interpreted as a boundary region with strategic design value. Once confusion is modeled in this way, architectural narrative selection becomes a problem of choosing an appropriate granularity: a description that is sufficiently novel to remain meaningful while also sufficiently comprehensible to support successful interpretation. In other words, the architectural task is not to eliminate uncertainty at all costs, but to manage uncertainty so that perceivers remain within a productive interpretive range. This perspective provides a stronger basis for narrative architectural design and more directly supports communication between architects and perceivers. In public cultural buildings, such improved interpretive accessibility contributes to human-centered sustainability in a cultural-social sense by strengthening public legibility, emotional identification, and long-term civic engagement rather than by directly addressing environmental performance.

The next sections are arranged in the following: Section 2 introduces the relevant concepts of three-way decisions theory and granular computing and clarifies the conceptual bridge across psychology, computation, and design. Section 3 introduces the interpretation method for the aesthetic model from the perspective of three-way decision. Section 4 presents the multi-granularity narrative cloud selection framework. Section 5 offers a case-based demonstration and a computer-simulated illustrative evaluation of candidate narrative statements. Section 6 discusses comparative positioning and practical applicability. Ultimately, Section 7 concludes the paper and outlines future empirical validation.

## 2 Preliminaries

### 2.1 Three-way decisions theory

Based on decision theory, the three-way decisions (3WD) theory was proposed by Yao (2010, 2011) from the perspective of decision risk. A domain is divided into three disjoint regions (positive region, negative region and boundary region) by computing the minimum decision risk in 3WD model. As shown in Figure 1, the rules generated from the positive region, negative region and boundary region represent the action of acceptance, rejection and deferment, respectively. In the view of cognitive science, the 3WD theory simulates the human thinking mechanism in solving problems. Recently, the concept of 3WD has attracted significant attentions in recent years, such as decision making (Liu et al., 2021), three-way decisions spaces (Hu, 2014), cognitive concept learning (Li et al., 2017) social networks (Jia et al., 2019), classification (Abdar et al., 2021), clustering (Khan et al., 2022) and recommendation (Ye and Liu, 2021).

**Figure 1:**
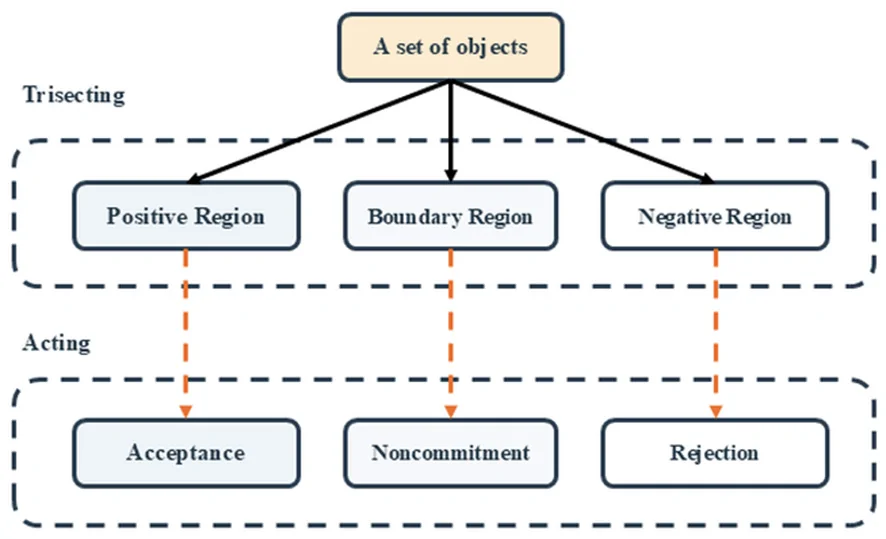
The diagram of 3WD classification.

3WD model brings new insight into the problem of parameter setting according to the minimum expected overall decision risk.

** Definition 1**. (Three-way decisions theory) (Yao, 2010) Given an decision system *S* = (*U, C*∪*D, V, f*), *R*⊆*C* and let *X* is a fuzzy set on *U*. The action set *A* = {*a*_*P*_, *a*_*B*_, *a*_*N*_} represents three kinds of actions which are acceptation, rejection and deferred decisions. λ_*PP*_, λ_*BP*_, λ_*NP*_ denote the losses incurred for taking actions*a*_*P*_, *a*_*B*_, *a*_*N*_, respectively. When an object belongs to target set *X*, and λ_*PN*_, λ_*BN*_, λ_*NN*_ denote the losses incurred for taking these actions when an object does not belong to target set *X*. Thus, the expected losses associated with taking different actions with object *x* can be expressed as follows,


$$\begin{array}{l} ℜ\left(a_{P}|\left[x\right]\right)=\lambda_{PP}\bar{\mu }\left(\left[x\right]\right)+\lambda_{PN}\left(1-\bar{\mu }\left(\left[x\right]\right)\right) \end{array}$$ (1)

$$\begin{array}{l} R\left(a_{B}|\left[x\right]\right)=\lambda_{BP}\bar{\mu }\left(\left[x\right]\right)+\lambda_{BN}\left(1-\bar{\mu }\left(\left[x\right]\right)\right) \end{array}$$ (2)

$$\begin{array}{l} R\left(a_{N}|\left[x\right]\right)=\lambda_{NP}\bar{\mu }\left(\left[x\right]\right)+\lambda_{NN}\left(1-\bar{\mu }\left(\left[x\right]\right)\right). \end{array}$$ (3)

Where, $\bar{\mu }\left(\left[x\right]\right)=\frac{\underset{y\subseteq \left[x\right]}{\sum }\mu \left(y\right)}{\left|\left[x\right]\right|}$ denotes the membership degree of the equivalence class [*x*] induced by equivalence relation *U*/*R* belong to object concept *X*, and $1-\bar{\mu }\left(\left[x\right]\right)$ denotes the membership degree of the equivalence class [*x*] induced by equivalence relation *U*/*R* belong to target set *X*^*C*^. From the perspective of probability and statistics, $\bar{\mu }\left(\left[x\right]\right)$ can be understood as the probability that a randomly selected object *x*∈[*x*] belong to target set *X*, and $1-\bar{\mu }\left(\left[x\right]\right)$ also can be understood as the probability that a randomly selected object *x*∈[*x*] not belong to target set *X*.

According to the Bayesian decision rule, the minimum-risk decision rules can be obtained as follows

(P) If *R*(*a*_*P*_|[*x*]) ≤ *R*(*a*_*N*_|[*x*])and *R*(*a*_*P*_|[*x*]) ≤ *R*(*a*_*B*_|[*x*]), decide *x*∈*POS*(*X*);

(B) If *R*(*a*_*B*_|[*x*]) ≤ *R*(*a*_*N*_|[*x*])and *R*(*a*_*B*_|[*x*]) ≤ *R*(*a*_*P*_|[*x*]), decide *x*∈*BND*(*X*);

(N) If *R*(*a*_*N*_|[*x*]) ≤ *R*(*a*_*B*_|[*x*])and *R*(*a*_*N*_|[*x*]) ≤ *R*(*a*_*P*_|[*x*]), decide *x*∈*NEG*(*X*).

(P) $\bar{\mu }\left(\left[x\right]\right)\geq \frac{\left(\lambda_{PN}-\lambda_{BN}\right)}{\left(\lambda_{PN}-\lambda_{BN}\right)+\left(\lambda_{BP}-\lambda_{PP}\right)}$ and $\bar{\mu }\left(\left[x\right]\right)\geq \frac{\left(\lambda_{PN}-\lambda_{NN}\right)}{\left(\lambda_{PN}-\lambda_{NN}\right)+\left(\lambda_{NP}-\lambda_{PP}\right)}$

(B) $\bar{\mu }\left(\left[x\right]\right)\leq \frac{\left(\lambda_{PN}-\lambda_{BN}\right)}{\left(\lambda_{PN}-\lambda_{BN}\right)+\left(\lambda_{BP}-\lambda_{PP}\right)}$and $\bar{\mu }\left(\left[x\right]\right)\geq \frac{\left(\lambda_{BN}-\lambda_{NN}\right)}{\left(\lambda_{BN}-\lambda_{NN}\right)+\left(\lambda_{NP}-\lambda_{BP}\right)}$

(N) $\bar{\mu }\left(\left[x\right]\right)\leq \frac{\left(\lambda_{PN}-\lambda_{NN}\right)}{\left(\lambda_{PN}-\lambda_{NN}\right)+\left(\lambda_{NP}-\lambda_{PP}\right)}$ and $\bar{\mu }\left(\left[x\right]\right)\leq \frac{\left(\lambda_{BN}-\lambda_{NN}\right)}{\left(\lambda_{BN}-\lambda_{NN}\right)+\left(\lambda_{NP}-\lambda_{BP}\right)}$

The decision rules can be re-expressed as follows:

(P) If $\bar{\mu }\left(\left[x\right]\right)\geq \alpha$and $\bar{\mu }\left(\left[x\right]\right)\geq \gamma$, decide *x*∈*POS*(*X*);

(B) If $\bar{\mu }\left(\left[x\right]\right)\leq \alpha$and $\bar{\mu }\left(\left[x\right]\right)\geq \beta$, decide *x*∈*BND*(*X*);

(N) If $\bar{\mu }\left(\left[x\right]\right)\leq \alpha$and $\bar{\mu }\left(\left[x\right]\right)\leq \gamma$, decide *x*∈*NEG*(*X*).

Combining with the above Equations 1–3, we can obtain the three parameters α, β, γ, respectively, according to the minimum-risk decision rules (P‘)-(N‘). The three decision thresholds are defined explicitly in Equations 4–6.

For rule(P′)


$$\begin{array}{l} \alpha =\frac{\left(\lambda_{PN}-\lambda_{BN}\right)}{\left(\lambda_{PN}-\lambda_{BN}\right)+\left(\lambda_{BP}-\lambda_{PP}\right)}={\left(1+\frac{\lambda_{BP}-\lambda_{PP}}{\lambda_{PN}-\lambda_{BN}}\right)}^{-1} \end{array}$$ (4)

For rule(B′)


$$\begin{array}{l} \beta =\frac{\left(\lambda_{BN}-\lambda_{NN}\right)}{\left(\lambda_{BN}-\lambda_{NN}\right)+\left(\lambda_{NP}-\lambda_{BP}\right)}={\left(1+\frac{\lambda_{NP}-\lambda_{BP}}{\lambda_{BN}-\lambda_{NN}}\right)}^{-1} \end{array}$$ (5)

For rule(N′)


$$\begin{array}{l} \gamma =\frac{\left(\lambda_{PN}-\lambda_{NN}\right)}{\left(\lambda_{PN}-\lambda_{NN}\right)+\left(\lambda_{NP}-\lambda_{PP}\right)}={\left(1+\frac{\lambda_{NP}-\lambda_{PP}}{\lambda_{PN}-\lambda_{NN}}\right)}^{-1} \end{array}$$ (6)

In other words, one can systematically determine the required threshold values from loss functions. In the present paper, the losses are assumed to be non-negative and order-constrained, namely 0 ≤ λ_*PP*_ ≤ λ_*BP*_ ≤ λ_*NP*_ and 0 ≤ λ_*NN*_ ≤ λ_*BN*_ ≤ λ_*PN*_, without restricting them to the unit interval. Under these assumptions, it still follows that 0 <α ≤ 1, 0 ≤ β ≤ 1, and 0 <γ ≤ 1. Furthermore, if $\frac{\lambda_{BP}}{\lambda_{NP}}+\frac{\lambda_{BN}}{\lambda_{PN}}\leq 1$, we have 0 ≤ β <γ <α ≤ 1 [25, 18]. The rules (P), (B), and (N) can be expressed as follows,

(P) if $\bar{\mu }\left(\left[x\right]\right)\geq \alpha$,decide $x\in POS_{R}^{\left(\alpha ,\beta \right)}\left(X\right)$;

(B) if $\beta \leq \bar{\mu }\left(\left[x\right]\right)<\alpha$, decide $x\in BND_{R}^{\left(\alpha ,\beta \right)}\left(X\right)$;

(N) if $\bar{\mu }\left(\left[x\right]\right)<\beta$, decide $x\in NEG_{R}^{\left(\alpha ,\beta \right)}\left(X\right)$.

### 2.2 Granular computing

Granular computing (GrC) (Yao et al., 2013; Yao, 2020; Wang and Li, 2020; Fu et al., 2020) is an umbrella term for a category of models inspired by human cognitive processes. There are three main GrC models: fuzzy sets (Zadeh, 1965), quotient space (Zhao and Zhang, 2006), and rough sets (Pawlak, 1984). Although each GrC model has its own method for solving problems, they have a common characteristic: a problem space will be divided into many subspaces that form a hierarchical structure before solving the problem. Granularity selection (Yang et al., 2019) is a prosperous issue in GrC, which aims to search an appropriate granularity to improve the efficiency of solving problems. At present, GrC has received much attention (Pedrycz et al., 2013; Loia et al., 2018; Li et al., 2022; Wan et al., 2021; Wang et al., 2017; Biederman and Vessel, 2006). Pedrycz et al. (2013); Loia et al. (2018) proposed the principle of justiable granularity by combining the construction and optimization method of granularity. Li et al. (2022) presented the granularity selection for the target concept and feature selection from the perspective of uncertainty based on these characteristic functions. Wan et al. (2021) introduced the idea of granularity of attributes is into triadic contexts on the basis of the relationship between triadic concept analysis and formal concept analysis. Wang et al. (2017) summarized the work of GrC from three aspects: granularity optimization, granularity switching and multi-granularity computing. In this paper, based on GrC theory, we will construct a multi-granularity narrative cloud selection method to improve the interpretation rate for the confusion state of population. This model is able to search for an optimal level of narrative cloud, which contributes to better understand the meaning of architecture and provides a more valuable basis for the subsequent narrative architectural design for designers.

### 2.3 The relationship among GrC, 3WD and cognitive science

A study of three-way decision and granular computing, in the context of results from cognitive science, is by itself an example of thinking in threes. Figure 2 shows the relationship among GrC, 3WD and cognitive science. For example, cognitive science provides a basis for three-way decision and the philosophy and methodology of three-way decision, as thinking in threes, can be used to study granular computing.

**Figure 2:**
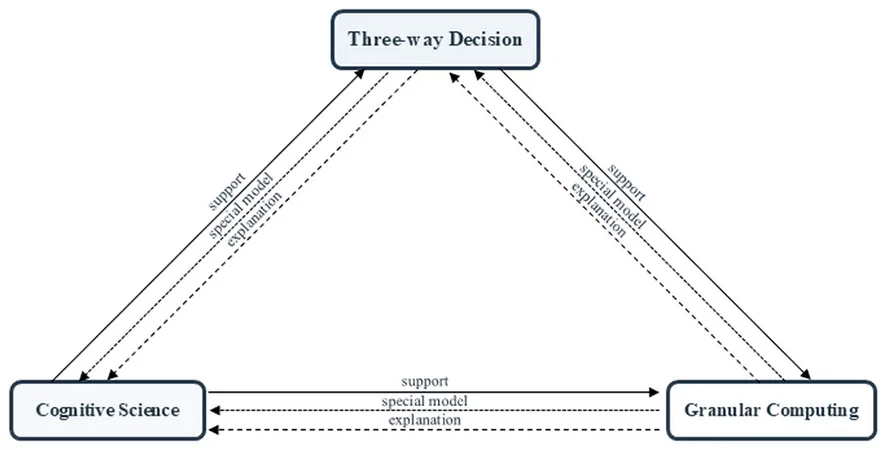
The relationship among GrC, 3WD, and cognitive science (Yao, 2020).

### 2.4 Conceptual bridge across psychology, computation, and design

To reduce conceptual jumps across disciplines, Table 1 summarizes how the main constructs used in this paper correspond to one another. The intention is not to claim that psychological states, decision-theoretic regions, and design operations are identical. Rather, the table clarifies how they can be linked in a design-support framework. In particular, confusion is treated as an interpretable uncertainty state: the user remains motivated, partial cues are available, and design intervention is still meaningful.

**Table 1:** Spatial element–narrative–perception correspondence in the memorial-hall example.

Architectural element	Narrative intent	Perceptible cue	Likely audience interpretation	Corresponding statement
Misaligned walls	Memory rupture	Interrupted sightlines and broken continuity	Historical discontinuity, uncertainty, rupture	*R*_12_
Rough fair-faced concrete	Endurance after suffering	Tactile roughness, gray tone, material heaviness	Resilience, trauma, sedimented time	*R*_22_
Dark corridor opening to courtyard	Rebirth and release	Abrupt light change and sudden spatial openness	Relief, hope, post-traumatic release	*R*_33_

## 3 Interpretation of PIA model in architecture based on 3WD theory

In the PIA model, the perceptual results of automatic processing are classified into positive emotion and negative emotion according to the availability of fluency, while the perceptual results of controlled processing are classified into disfluency reduction (Yes DR), no disfluency reduction (No R), and no disfluency reduction (No D). Among these outcomes, Yes DR is associated with a positive aesthetic response, whereas the other two outcomes are usually associated with negative responses. Confusion is especially relevant here because the perceiver may still be motivated to invest cognitive effort, even though the stimulus has not yet produced sufficient progress in meaning construction or fluency increment.

In this paper, based on the idea of three-way decision (3WD) theory, we interpret the PIA model in architecture for people in confused state of controlled processing. To make the discussion easier to follow from a design perspective, the three states are described here through typical audience situations. The interest state refers to users who can connect spatial cues with intended meaning after a manageable amount of cognitive effort; for example, a design-trained visitor may understand that an intentionally broken circulation sequence symbolizes historical rupture and therefore experience interpretive reward. The confusion state refers to users who sense that the building is meaningful but cannot yet stabilize an interpretation; for example, a general visitor may notice the unusual path, tilted wall, and abrupt light transition, but may still be unsure whether these cues represent memory, disaster, resistance, or some other narrative. The negative or rejection state refers to users for whom the semantic distance remains too large; in that situation, the architectural message is not decoded and the visitor is more likely to abandon interpretation or reduce the building to a purely formal impression.

** Example 1**. As shown in Table 2, suppose that a memorial-type building contains 500 units of architectural information perceived by five representative audience groups. These groups may be understood as architecture students, art enthusiasts, local residents, the lead architect, and general tourists. The amounts of information perceived by the five groups are 50, 50, 100, 150 and 150, respectively. Let *C* denote the subset of 285, that is, the total amount of information that is successfully interpreted is 285.

**Table 2:** Conceptual bridge among PIA constructs, 3WD regions, observable indicators, and design interventions.

PIA construct	Cognitive meaning	3WD mapping	Observable indicator	Design intervention
Interest	Willing and able to interpret	Positive region	High comprehension and high interpretive interest	Preserve complexity and maintain meaningful challenge
Confusion	Motivated but not yet able to stabilize interpretation	Boundary region	Medium comprehension, high exploratory intention, partial semantic grasp	Adjust narrative granularity and provide intermediate interpretive scaffolds
Boredom	Low novelty or low motivational engagement	Not directly equivalent to 3WD negative region	Low interest, low cognitive investment, rapid dismissal	Increase novelty, reorganize cues, or enrich the experiential sequence
Rejection	Semantic distance too large for current interpretation	Negative region	Low comprehension and abandonment of interpretive effort	Provide basic guidance or reconstruct spatial and symbolic cues

Where, *C*∩*E* denotes the number in the corresponding set (the amount of information successfully interpreted). *C*^*C*^∩*E* denotes the amount of information that was not successfully interpreted. *pr*(*C*|*E*)and *pr*(*C*^*C*^|*E*)denotes the conditional probability of interpreting the amount of successful information in the corresponding set, respectively. This design-oriented grouping is useful because it translates the abstract equivalence classes of 3WD into recognizable audience states. In practice, the architect is expected to occupy the most interpretable position, students and trained viewers often remain near the interest state, local residents and culturally engaged visitors frequently enter the confusion state, and general tourists are more likely to fall into rejection when the narrative remains too implicit.

The following loss values are used as illustrative parameters for demonstrating the decision logic of the framework. They are not intended as empirically calibrated constants. Rather, they express an asymmetric design situation in which falsely assuming successful interpretation, prematurely rejecting potentially interpretable users, and delaying decision for additional narrative support have different consequences.

Set the loss function as follows:

λ_*PP*_ = 0, λ_*PN*_ = 17, λ_*BP*_ = 9, λ_*BN*_ = 2, λ_*NP*_ = 15, λ_*NN*_ = 0,

Then,


$$\begin{array}{l} \alpha =\frac{\left(\lambda_{PN}-\lambda_{BN}\right)}{\left(\lambda_{PN}-\lambda_{BN}\right)+\left(\lambda_{BP}-\lambda_{PP}\right)}={\left(1+\frac{\lambda_{BP}-\lambda_{PP}}{\lambda_{PN}-\lambda_{BN}}\right)}^{-1}\\ \beta =\frac{\left(\lambda_{BN}-\lambda_{NN}\right)}{\left(\lambda_{BN}-\lambda_{NN}\right)+\left(\lambda_{NP}-\lambda_{BP}\right)}={\left(1+\frac{\lambda_{NP}-\lambda_{BP}}{\lambda_{BN}-\lambda_{NN}}\right)}^{-1}\\ \gamma =\frac{\left(\lambda_{PN}-\lambda_{NN}\right)}{\left(\lambda_{PN}-\lambda_{NN}\right)+\left(\lambda_{NP}-\lambda_{PP}\right)}={\left(1+\frac{\lambda_{NP}-\lambda_{PP}}{\lambda_{PN}-\lambda_{NN}}\right)}^{-1} \end{array}$$

We have α = 0.63, β = 0.25, γ = 0.53

The positive region, boundary region and negative region are calculated as follows:

Pawlak model:


$$\begin{array}{l} POS_{\left(1,0\right)}\left(C\right)=E_{4}\\ BND_{\left(1,0\right)}\left(C\right)=E_{1}\cup E_{2}\cup E_{3}\cup E_{5}\\ NEG_{\left(1,0\right)}\left(C\right)=\emptyset \end{array}$$

Three-way model:


$$\begin{array}{l} POS_{\left(0.63,0.25\right)}\left(C\right)=E_{1}\cup E_{4}\\ BND_{\left(0.63,0.25\right)}\left(C\right)=E_{2}\cup E_{3}\\ NEG_{\left(0.63,0.25\right)}\left(C\right)=E_{5} \end{array}$$

par Two-way model:


$$\begin{array}{l} POS_{\left(0.53,0.53\right)}\left(C\right)=E_{1}\cup E_{3}\cup E_{4}\\ BND_{\left(0.53,0.53\right)}\left(C\right)=\emptyset \\ NEG_{\left(0.53,0.53\right)}\left(C\right)=E_{2}\cup E_{5} \end{array}$$

It can be seen that the boundary of two-way model is always the empty set.

From a design interpretation perspective, the three-way result is the most meaningful. *E*_1_ and *E*_4_ fall into the positive region because these groups already possess enough disciplinary knowledge or direct authorial intention to transform challenging spatial cues into interest. *E*_2_ and *E*_3_ fall into the boundary region because they do not fully fail; rather, they partially understand the architectural message and therefore represent the most valuable target population for narrative assistance. In practical terms, these users are the ones who would benefit most from a better calibrated narrative layer, for example, a more accessible exhibition text, a clearer interpretive sequence, or a more legible material-symbolic cue. *E*_5_ falls into the negative region because the semantic distance is too large and the design, without additional support, is unlikely to produce stable meaning acquisition. From Table 3, for the equivalence class *E*_1_: the Pawlak model makes delayed decisions with a misclassification cost as follows:


$$\begin{array}{l} cost^{p}=\lambda_{BP}\mathrm{Pr}\left(C|E_{1}\right)+\lambda_{BN}\mathrm{Pr}\left(C^{c}|E_{1}\right)\\ \;=9*0.80+2*0.20=7.60 \end{array}$$

**Table 3:** Information table.

Audience group (equivalence class)	*C*∩*E*	*C*^*C*^∩*E*	*pr*(*C*|*E*)	*pr*(*C*^*C*^|*E*)
*E*_1_ (architecture students, 50)	40	10	0.8	0.2
*E*_2_ (art enthusiasts, 50)	20	30	0.4	0.6
*E*_3_ (local residents, 100)	60	40	0.6	0.4
*E*_4_ (lead architect, 150)	150	0	1	0
*E*_5_ (general tourists, 150)	15	135	0.1	0.9

In contrast, both the two-way and three-way models made acceptance decisions. The associated misclassification cost are given by the following equation:


$$\begin{array}{l} cost^{b}=cost^{t}=\lambda_{PP}\mathrm{Pr}\left(C|E_{1}\right)+\lambda_{PN}\mathrm{Pr}\left(C^{c}|E_{1}\right)\\ \;=0*0.80+17*0.20=3.40 \end{array}$$

Therefore, the acceptance decision suggested by the three-way model has a low cost. Using similar calculations, Table 4 summarizes the decisions and associated costs for all equivalence classes.

**Table 4:** The comparison of the three decision models.

Audience group (equivalence class; n)	Pawlak model	Three-way model	Two-way model
*E*_1_(50)	Deferment: 7.60	Acceptance: 3.40	Acceptance: 3.40
*E*_2_(50)	Deferment: 4.80	Deferment: 4.80	Rejection: 6.00
*E*_3_(100)	Deferment: 6.20	Deferment: 6.20	Acceptance: 6.80
*E*_4_(150)	Acceptance: 0.00	Acceptance: 0.00	Acceptance: 0.00
*E*_5_(150)	Deferment: 2.70	Rejection: 1.50	Rejection: 1.50

Obviously, the misclassification cost of the three-way model is always the same as the lower cost of the Pawlak and two-way models. For the Pawlak model the overall decision data are as follows.


$$\begin{array}{l} \begin{array}{l} Cost^{p}=\frac{1}{1000}[\lambda_{PP}n_{PP}^{p}+(\lambda_{BP}n_{BP}^{p}+\lambda_{BN}n_{BN}^{p})+\lambda_{NN}n_{NN}^{p}]\\ =\frac{1}{1000}[0*300+(9*270+2*430)+0*0]=3.29 \end{array}\\ \begin{array}{l} Cos{\text{t}}^{t}=\frac{1}{1000}[\left(\lambda_{PP}n_{PP}^{t}+\lambda_{PN}n_{PN}^{t}\right)]\\ \left[+(\lambda_{BP}n_{BP}^{t}+\lambda_{BN}n_{BN}^{t})+(\lambda_{NP}n_{NP}^{t}+\lambda_{NN}n_{NN}^{t})\right]\\ =\frac{1}{1000}[(0*380+17*20)+(9*160+2*140)\\ +(15*30+0*270)]=2.51 \end{array}\\ \begin{array}{l} Cost^{b}=\frac{1}{1000}[\left(\lambda_{PP}n_{PP}^{b}+\lambda_{PN}n_{PN}^{b})+(\lambda_{NP}n_{NP}^{b}+\lambda_{NN}n_{NN}^{b}\right)]\\ =\frac{1}{1000}[\left(0^{*}500+17*100)+(15*70+0*330\right)]=2.75 \end{array} \end{array}$$

The strategy based on the arithmetic available 3WD is more advantageous in dealing with the three states of the PIA model. Based on the above results, this paper emphasizes the importance of the confusion boundary state in architectural design. Due to the unique advantages of narrative architecture, the aim is to guide the perception of users who remain motivated and possess partial interpretive ability, but still require better calibrated semantic cues. The scientific design of narratives in architecture is therefore the central concern of this paper.

## 4 A multi-granularity narrative cloud selection integrating aesthetic model

An obvious characteristic of human intelligence is to deal with problems hierarchically. In other words, complex problems are usually analyzed at different levels of granularity rather than on a single descriptive layer. Granularity reflects the degree of detail used to represent information, and when the problem is complex, coarse and fine descriptions should be coordinated to satisfy practical constraints of interpretation and decision making. In architectural aesthetics, although many studies have explored the communication distance between designers and perceivers, it remains difficult to identify a narrative form that is simultaneously understandable, meaningful, and sufficiently novel for most users. Prior cognitive and neuroaesthetic studies have shown that informational reward is associated with pleasurable perception, especially when the stimulus offers both learning potential and interpretable structure (Biederman and Vessel, 2006; Yue et al., 2007). Recent built-environment research likewise suggests that preference, attention, restorative response, and perceived environmental quality depend on how environmental information is organized and interpreted by users (Liu and Guo, 2024; Son and Kim, 2024; Deng et al., 2026; Thampanichwat et al., 2024; Wu et al., 2024; Ji and Li, 2024; Bunyarittikit et al., 2026). Therefore, based on GrC theory, we propose a multi-granularity narrative cloud selection method to identify descriptions that are both novel and understandable for people in the confusion state. In this paper, novelty (*NO*) and comprehensibility (*EU*) are treated as normalized evaluation variables in the range [0, 1]. In the illustrative case, these values are generated through a computer-simulated scoring procedure to demonstrate the workflow of the framework. In practical applications, however, the same variables can be obtained through reproducible user ratings, expert assessments, semantic differential scales, Likert-type questionnaires, structured interviews, or controlled perception experiments. For example, target users or domain experts may rate each candidate narrative statement in terms of perceived novelty and comprehensibility; the raw scores can then be averaged and normalized to [0, 1] before being entered into the selection model. The following heuristic utility function is established to search for the balance between novelty and comprehensibility. The utility of each granular layer is calculated using Equation 7.


$$\begin{array}{l} U_{NC_{i}}=\theta NO_{NC_{i}}+\left(1-\theta \right)EU_{NC_{i}} \end{array}$$ (7)

Where, *U*_*N*_*C*__*i*__ denotes the heuristic utility of granular layer *NC*_*i*_, and θ∈[0, 1] reflects the user's relative preference for novelty and comprehensibility. A larger value of *U*_*N*_*C*__*i*__ indicates a more desirable balance between cognitive accessibility and semantic tension. In light of the inverted-U relationship discussed in neuroaesthetics (Biederman and Vessel, 2006; Yue et al., 2007), the narrative cloud with the highest comprehensibility alone is not necessarily the best narrative cloud. In practical architectural communication, the preferred description should retain enough novelty to sustain interpretive interest while remaining understandable to the target audience. The parameter θ can be adjusted according to design goals and user groups. A memorial or museum may assign a higher weight to novelty when reflective engagement is desired, whereas healthcare, transportation, or other high-stress public settings may assign a higher weight to comprehensibility in order to reduce cognitive burden. In applied projects, θ should ideally be set through expert discussion, pilot rating, or iterative calibration with target users.

If the perceivers' requirements for novelty and comprehensibility are denoted by *NO*_*user*_ and *EU*_*user*_ respectively, the narrative cloud optimization aims to search a narrative cloud *NC*_*i*_ that meets both conditions *NO*_*N*_*C*__*i*__≥*NO*_*user*_ and *EU*_*N*_*C*__*i*__≥*EU*_*user*_, and then express the description using this narrative cloud. As shown in Figure 3, this is the multi-granularity optimization mechanism for the narrative cloud. Herein, narrative cloud *NC*_*r*_ meets the novelty requirement but fails to meet the comprehensibility requirement. *NC*_*s*_ meets the comprehensibility requirement but fails to meet the novelty requirement. *NC*_*t*_ meets the requirements for both novelty and comprehensibility. This optimization mechanism can be expressed as the following formula. The resulting constrained optimization problem is stated in Equation 8.


$$\begin{array}{l} \arg\max U_{NC_{i}} \end{array}$$ (8)

$$\begin{array}{l} s.t.NO_{NC_{i}}\geq NO_{user},EU_{NC_{i}}\geq EU_{user} \end{array}$$

**Figure 3:**
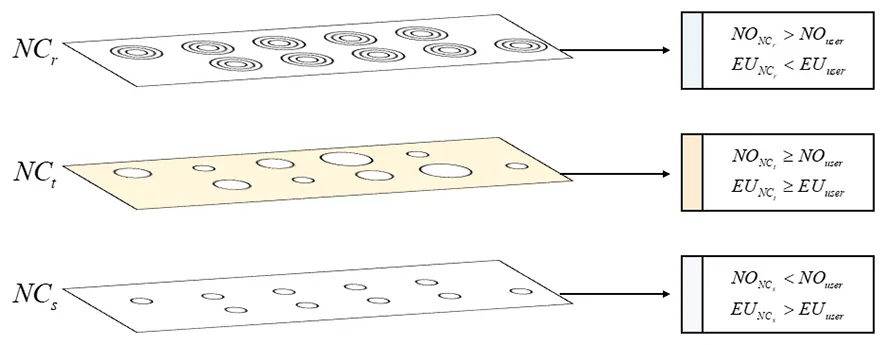
The optimal granularity selection based on GrC.

Figure 4 shows the architectural design process, which is defined in detail as follows: (1) the architect brainstorms a narrative cloud about the theme (the architect should expand the scope of the narrative and also involve different architects or target perceivers of the building); (2) the statements in the narrative cloud are classified (the classification is based on the theme to determine the classification criteria); (3) researching the classified statements for the target audience of the building (based on novelty and comprehensibility); (4) determining the best combination of statements in each category to form the narrative cloud, and (5) designing the building according to the narrative cloud. Throughout the process, the narrative cloud is categorized in order to avoid possible errors in the research under different categories and to be able to describe the building more comprehensively.

**Figure 4:**
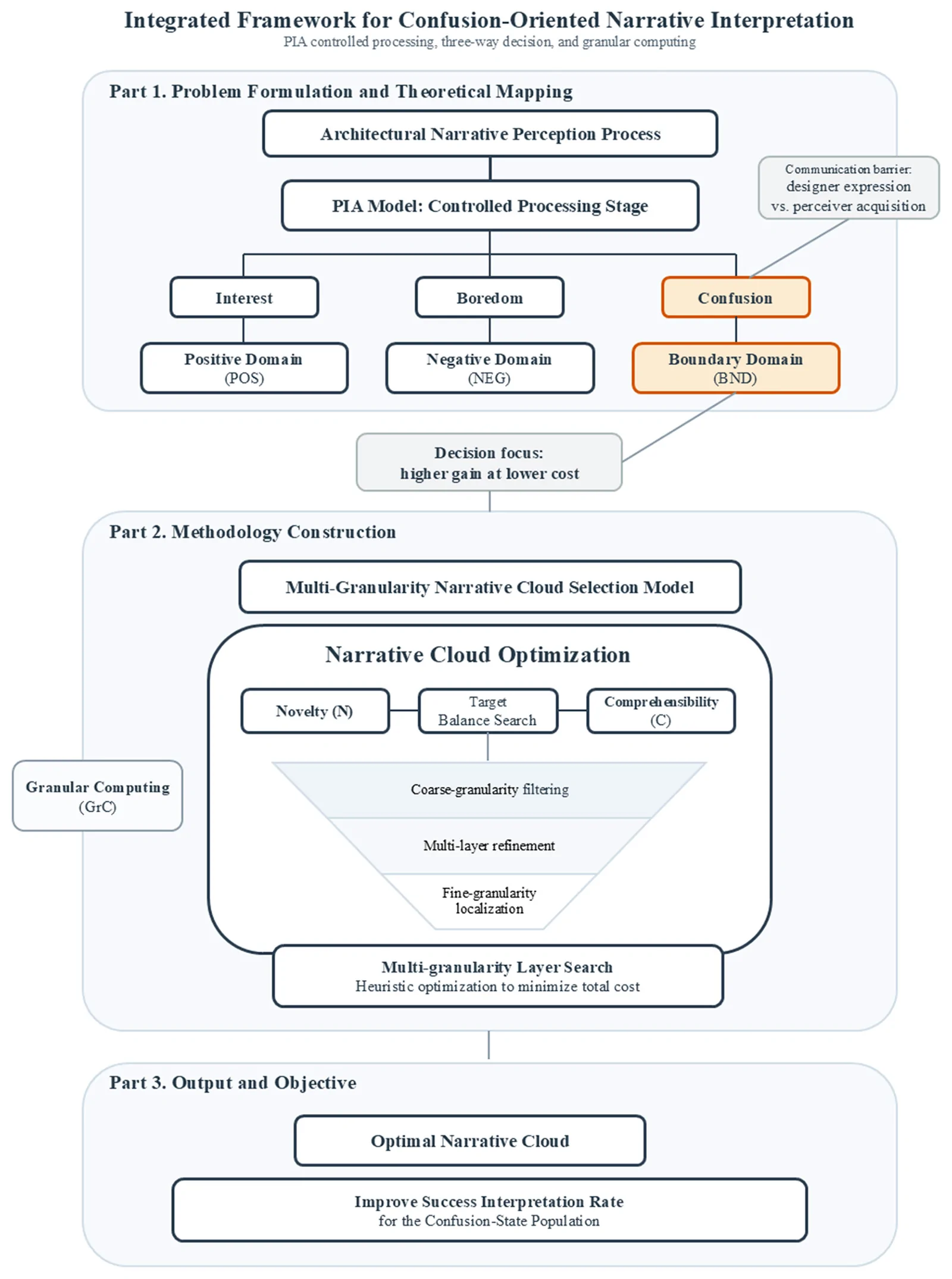
The process of architectural design.

## 5 Case-based demonstration and illustrative evaluation workflow

To acquire the optimal granularity for statement selection under the above constraints, we present an explicit search procedure for identifying the most suitable candidate in each narrative dimension. The overall procedure should be understood as a two-stage operationalization of the theoretical framework. In the first stage, the PIA model and three-way decision theory identify the interpretive state of the target audience. The thresholds α, β, and γ define whether a user group is located in the positive region, boundary region, or negative region. The boundary region corresponds to confusion-state users who retain interpretive motivation but have not yet achieved stable meaning-making. In the second stage, Algorithm 1 is applied to this boundary-region audience to select narrative statements by balancing novelty and comprehensibility. Thus, α, β, and γ do not function as direct utility weights in Algorithm 1; rather, they determine the decision context and the target population for which narrative-cloud optimization is required. The procedure is framed as a heuristic utility-maximization problem rather than as a fully validated prediction model. It therefore serves as an illustrative design-support algorithm that can be implemented and empirically refined in future work.

Algorithm 1 Heuristic selection of narrative statements based on GrC. 
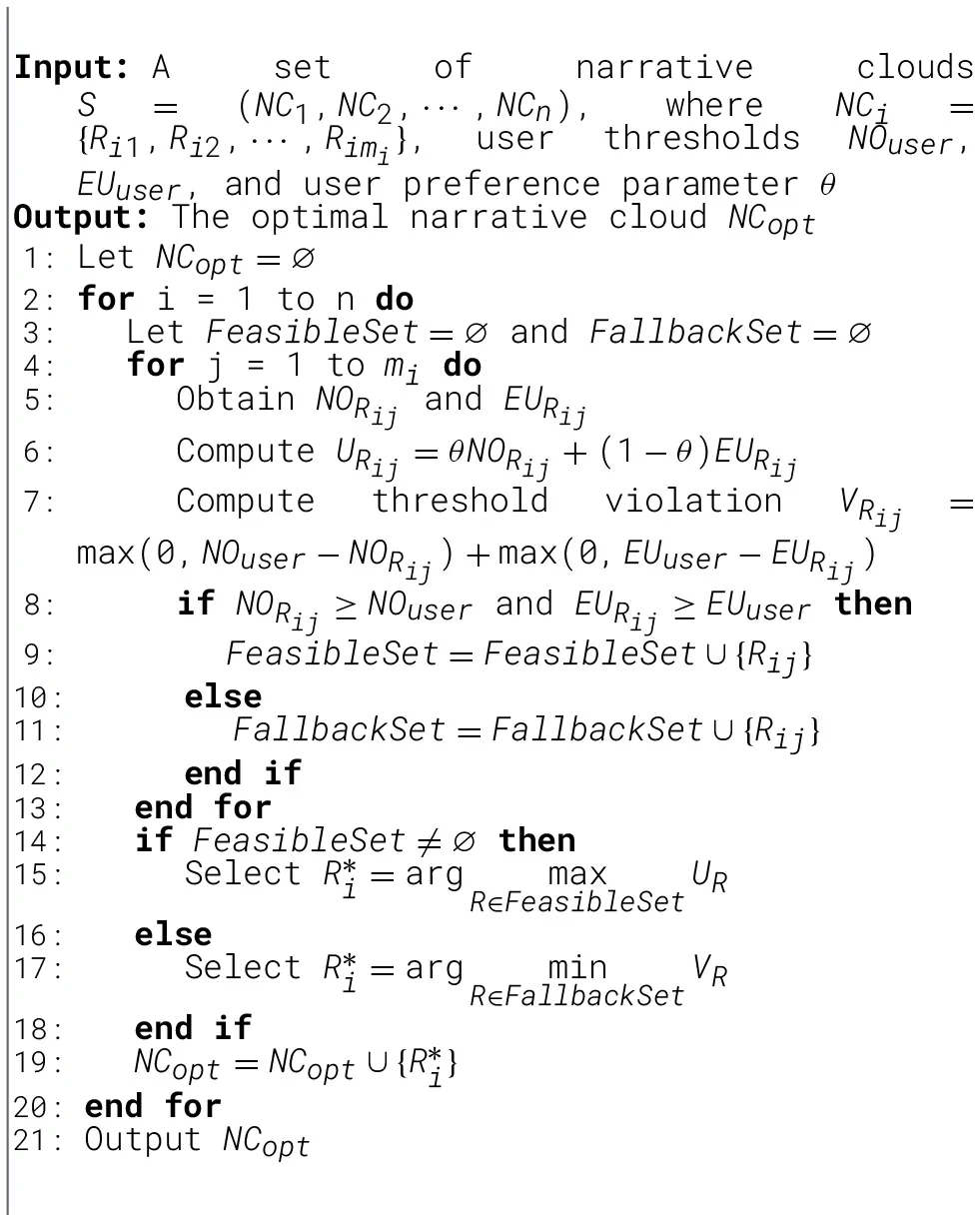

In the following, the procedure is illustrated through a design-oriented memorial-hall example together with a computer-simulated illustrative scoring exercise.

** Example 2**. Suppose that a city plans to build a memorial hall with the theme of “memory and rebirth” on a post-war ruin site. The project adopts fragmented geometry, non-linear circulation, rough material transitions, and abrupt changes in light in order to metaphorize rupture, resilience, and temporal transformation. Here, the memorial hall should be understood as an illustrative design-oriented case rather than as a completed empirical validation. A set of narrative cloud *S* = (*NC*_1_, *NC*_2_, *NC*_3_) is generated for describing the building, where *NC*_1_ = (*R*_11_, *R*_12_, *R*_13_), *NC*_2_ = (*R*_21_, *R*_22_), and *NC*_3_ = (*R*_31_, *R*_32_, *R*_33_, *R*_34_). To make the computational procedure explicit, novelty and comprehensibility values are treated as computer-simulated illustrative scores and then normalized to the interval [0, 1]. These simulated scores are used to demonstrate how the proposed framework operates under novelty and comprehensibility constraints; they should not be interpreted as empirical user-rating data. We assume that θ = 0.5, which means that the user's preference for novelty and comprehensibility is balanced. In addition, the user's requirements are set as *NO*_*user*_ = 0.2 and *EU*_*user*_ = 0.1. In this application, the task is to search for the optimal statement in each narrative dimension and then combine these statements into a new narrative cloud, denoted by *NC*_*opt*_.

Table 1 links the narrative statements to spatial elements, perceptible cues, and likely audience interpretations, thereby clarifying that the framework is not limited to explanatory text alone. Rather, the narrative cloud may include concept narrative, spatial narrative, material narrative, path narrative, symbolic narrative, exhibition narrative, guide-text narrative, signage, wayfinding support, digital guidance, and post-design public interpretation. Table 5 shows the illustrative results obtained by the proposed heuristic procedure using computer-simulated illustrative scores. Furthermore, Figures 5, 6 are presented to describe the results more intuitively. Figure 5 shows the novelty, comprehensibility, and utility of each statement. Figure 6 shows utility under different novelty and comprehensibility combinations.

**Table 5:** Multi-granularity evaluation of narrative statements in the memorial-hall case.

Dimension	Code	Narrative statement	Novelty	Compreh.	Utility	Decision
*NC*_1_	*R*_11_	The folded non-linear skin directly imitates fragments of historical ruin topology.	0.07	0.27	0.17	No
*NC*_1_	*R*_12_	Misaligned walls cut sightlines, metaphorizing the discontinuity of memory across time.	0.21	0.11	**0.16**	Yes
*NC*_1_	*R*_13_	Spatial boundaries are intentionally blurred to represent the chaotic condition of disaster.	0.15	0.13	0.14	No
*NC*_2_	*R*_21_	The large stair rising through the building resembles roots growing upward from the ground, suggesting resilience and life force.	0.23	0.12	**0.175**	Yes
*NC*_2_	*R*_22_	The rough texture of fair-faced concrete conveys an attitude of endurance after turmoil.	0.18	0.22	0.20	No
*NC*_3_	*R*_31_	Moving skylight patches form a silent temporal scale on the wall surface.	0.17	0.25	0.21	No
*NC*_3_	*R*_32_	A repeatedly winding route stretches the visitor's sense of time and slows down bodily pace.	0.22	0.09	0.155	No
*NC*_3_	*R*_33_	The sudden transition from a dim corridor to an open courtyard stages a temporal leap from oppression to release.	0.21	0.15	**0.18**	Yes
*NC*_3_	*R*_34_	Rusted steel plates and transparent glass symbolize a trans-temporal dialogue between past and present.	0.25	0.09	0.17	No

Bold values indicate the utility scores of the statements selected for the optimal narrative cloud after application of the novelty and comprehensibility thresholds within each narrative dimension.

**Figure 5:**
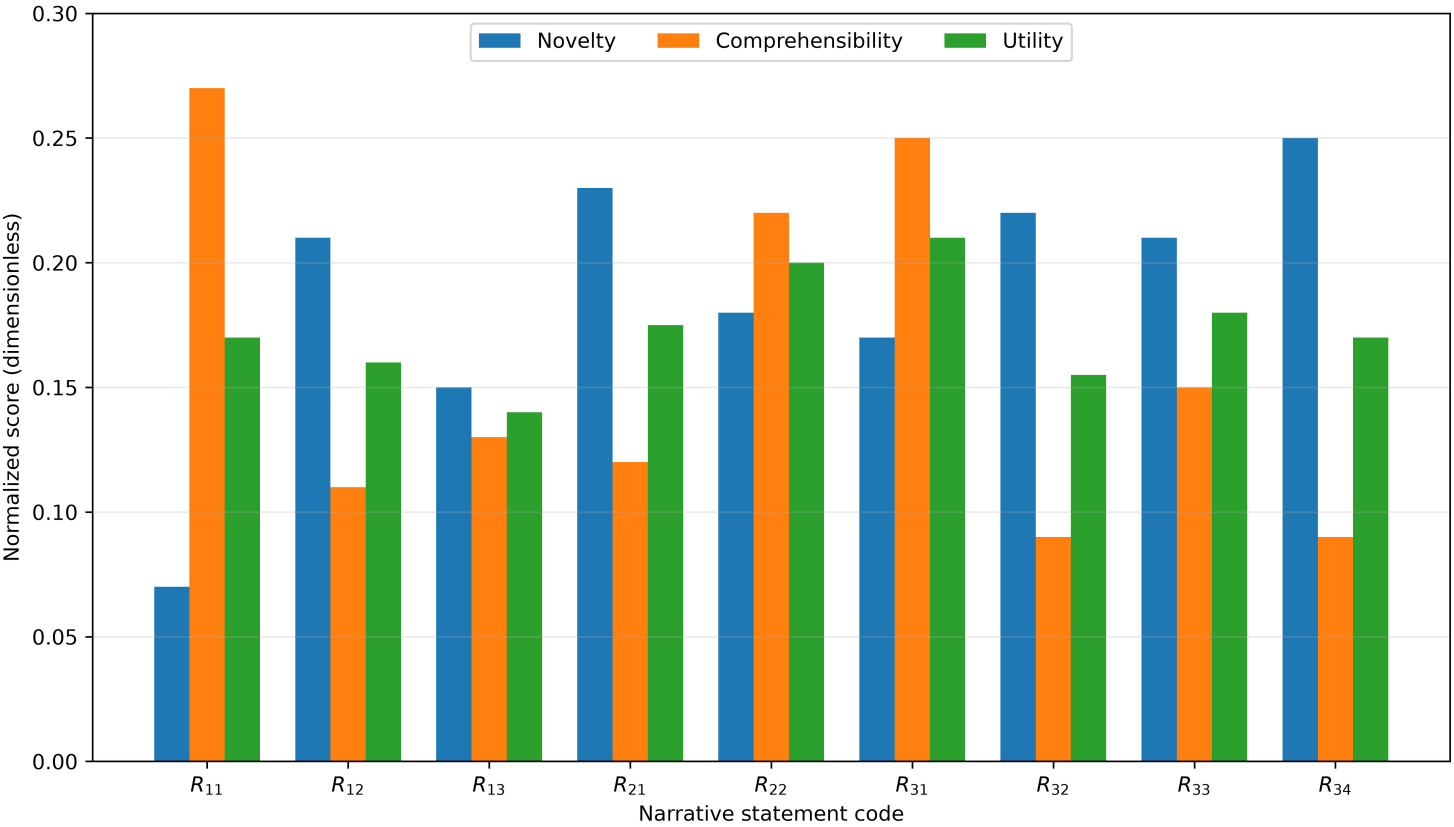
Novelty, comprehensibility, and utility of the candidate narrative statements.

**Figure 6:**
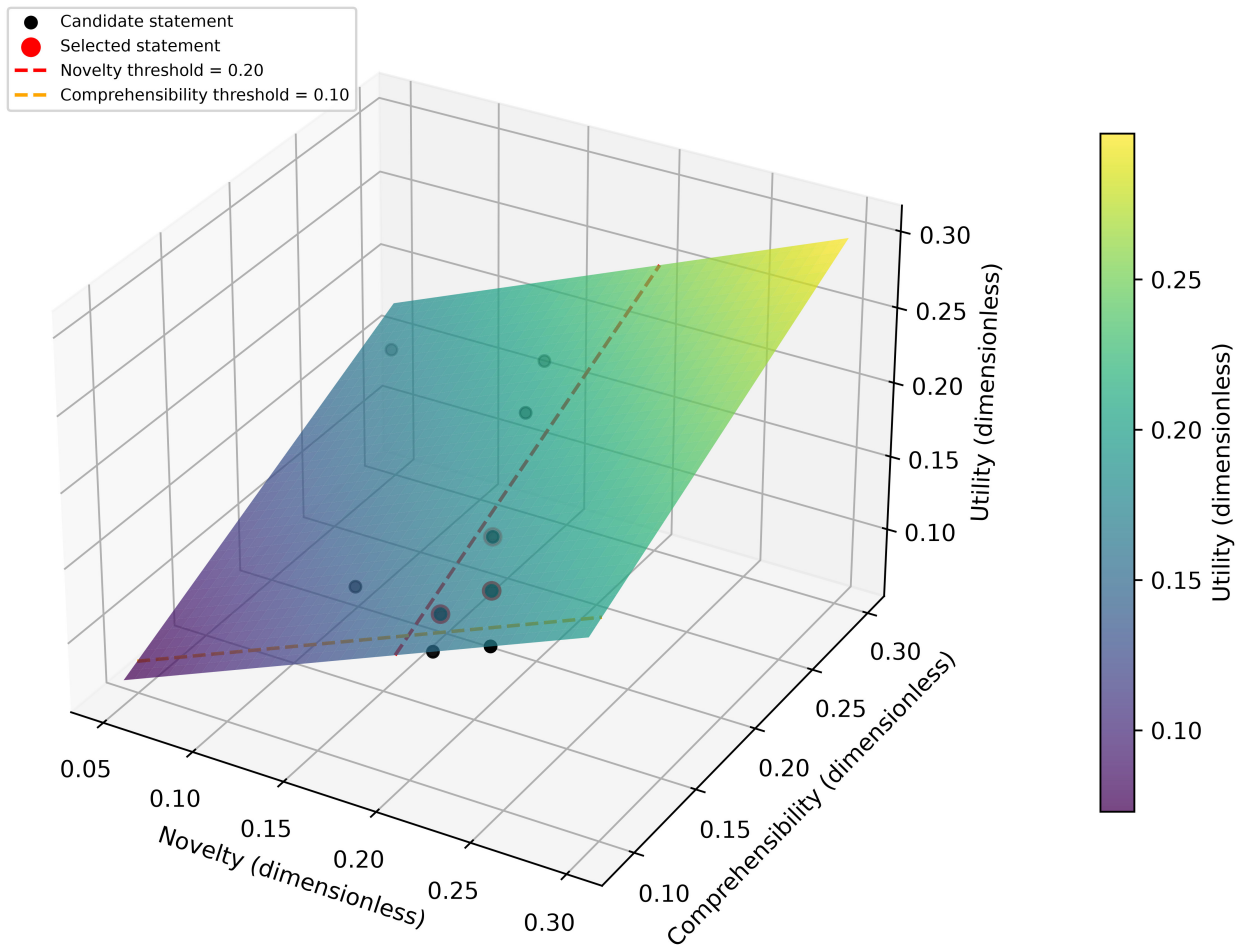
Utility surface under different novelty and comprehensibility combinations.

From these results, it can be seen that the algorithm does not simply prefer the most understandable or the most novel description. Rather, it filters out statements that are too ordinary to sustain interpretive interest and also excludes statements that are too obscure to support comprehension. The final optimal narrative cloud is *NC*_*opt*_ = {*R*_12_, *R*_21_, *R*_33_}.

This result can be interpreted from a design perspective. *R*_11_ is easy to process but lacks semantic tension, and therefore it fails to activate sufficient interpretive curiosity. *R*_22_, although relatively understandable, does not satisfy the novelty threshold and is therefore excluded under the current settings. *R*_34_ is highly novel but falls below the comprehensibility threshold; if imposed on a confusion-state audience, it is likely to push that audience toward frustration rather than toward meaningful engagement. By contrast, *R*_12_, *R*_21_, and *R*_33_ all preserve a clear phenomenological anchor in the physical environment—walls, stairs, and light-space transition—while still leaving enough poetic openness for users to construct meaning. In this sense, the algorithm identifies not merely an optimal statement set, but an optimal design scaffold for people located in the confusion boundary region.

## 6 Comparative positioning and practical applicability

The proposed framework is not intended to replace architectural judgment, cultural interpretation, or participatory design. Its purpose is to provide a structured decision-support procedure for organizing candidate narrative statements and identifying a suitable balance between novelty and comprehensibility for users who remain in the confusion boundary region. Table 6 summarizes the qualitative difference between the proposed framework and several common approaches to architectural narrative selection.

**Table 6:** Qualitative comparison with common narrative selection approaches.

Approach	Main basis	Limitation	Difference of the proposed framework
Designer intuition	Authorial intention and design experience	May overlook heterogeneous audience interpretation	Explicitly considers target users in the confusion boundary region
Expert-led interpretation	Professional knowledge and curatorial judgment	May remain difficult for non-specialist users	Balances semantic tension with cognitive accessibility
Post-design explanation	Exhibition text, guide text, signage, or public interpretation	Often added after spatial decisions have been fixed	Can inform both design communication and narrative organization during design
Simple single-criterion selection	Highest clarity, highest novelty, or strongest symbolic expression	May produce statements that are too obvious or too obscure	Uses a dual-constraint utility model combining novelty and comprehensibility

In practical terms, the framework can be adapted to several architectural contexts. In museums and exhibition buildings, it may support the selection of interpretive routes, display texts, symbolic cues, and spatial sequences. In educational buildings, it may help align spatial narratives with institutional memory, learning identity, and collective belonging. In healthcare facilities, the framework should be used more cautiously, with greater weight given to comprehensibility, emotional comfort, and reduced cognitive load. In public spaces, it may support wayfinding, civic interpretation, cultural memory, and inclusive communication for users with different levels of background knowledge. Across these contexts, the values of *NO*, *EU*, *NO*_*user*_, *EU*_*user*_, and θ should not be treated as universal constants. They should be selected according to the building type, target audience, narrative goal, and design stage, and should preferably be calibrated through expert discussion, small pilot evaluation, or user-centered semantic assessment.

## 7 Conclusion

In this paper, we reinterpret the three perceived outcomes of the controlled-processing phase of the PIA model from the perspective of three-way decision theory and show the design value of the confusion state. Rather than treating confusion as a purely negative endpoint, the proposed framework models it as a boundary region in which design intervention remains meaningful and potentially valuable. From the viewpoint of architectural communication, this is important because many perceivers possess sufficient motivation and partial interpretive ability, but still encounter barriers between the architect's intended meaning and the meaning that can actually be acquired from the built form.

On this basis, a multi-granularity narrative cloud selection method is constructed to support architectural interpretation among confusion-state users while preserving the novelty required for architectural meaning-making. By utilizing granular computing theory, the proposed method searches for an appropriate level of narrative description that balances comprehensibility and novelty and therefore provides a more rigorous basis for subsequent narrative architectural design. More broadly, the study contributes to current research on neuroaesthetics, architectural cognition, and human-centered building design by clarifying how meaning-oriented design decisions may be linked to interpretable uncertainty. The memorial-hall example and computer-simulated scoring exercise illustrate the feasibility of this framework, but they do not constitute full empirical validation. A small pilot evaluation with target users or domain experts would be a necessary next step for testing whether the selected narrative statements are perceived as intended. Future work should therefore further test and refine the framework through real-case applications, user studies, expert assessment, eye-tracking, immersive environments, physiological measures, and user-based semantic evaluation.
